# Predictors and Patterns of Recurrence After a Watchful Waiting Approach following Clinical Complete Response to Neoadjuvant Radiochemotherapy for Esophageal Cancer

**DOI:** 10.3390/curroncol33030170

**Published:** 2026-03-16

**Authors:** Sarah Gerber, Martin D. Berger, Hossein Hemmatazad, Pauline Aeschbacher, Dino Kröll, Daniel Candinas, Yves Borbély

**Affiliations:** 1 Department of Visceral Surgery and Medicine, Inselspital, Bern University Hospital, University of Bern, 3010 Bern, Switzerland; 2 Department of Medical Oncology, Inselspital, Bern University Hospital, University of Bern, 3010 Bern, Switzerland; 3 Department of Radiation Oncology, Inselspital, Bern University Hospital, University of Bern, 3010 Bern, Switzerland

**Keywords:** esophageal cancer, watchful waiting, organ sparing approach, esophagus, tumor recurrence

## Abstract

(1) Background: Treatment of esophageal cancer (EC) traditionally consists of neoadjuvant radiochemotherapy (RCT) followed by resection; however, esophagectomy is associated with substantial morbidity, particularly in patients with relevant comorbidities. Therefore, a watchful waiting (WW) strategy has been increasingly adopted for patients achieving a complete response to RCT. This study aimed to identify independent predictors and recurrence patterns in EC patients managed with WW. (2) Methods: We retrospectively analyzed all patients with potentially curable EC and complete response to RCT treated at a tertiary university hospital between 2014 and 2022. Comprehensive staging and restaging were performed using computed tomography, endoscopy with ultrasound and biopsies, and positron-emission tomography, followed by structured surveillance. Recurrence patterns and associated clinical and tumor-related factors were assessed using multivariate regression analysis. (3) Results: Among 50 included patients, 30 (60%) developed recurrence after a median of 202 days. Patients with initially nodal-negative disease did not develop distant recurrence, whereas nodal-positive patients showed metastatic recurrence in 26% and local regrowth in 16%. (4) Discussion: Adenocarcinoma, circumferential tumor extent greater than 50%, dysphagia, fatigue, and deterioration of general condition at restaging were independently associated with recurrence. These findings support risk-adapted surveillance and may facilitate personalized management in EC patients undergoing WW.

## Simple Summary

Esophageal cancer is usually treated with chemotherapy and radiation followed by surgery, but surgery can be risky and cause serious complications. For some patients who respond completely to initial treatment, a “watchful waiting” approach—delaying or avoiding surgery unless the cancer returns—is increasingly considered. In this study, we analyzed patients managed with watchful waiting to identify factors linked to cancer recurrence. We found that having adenocarcinoma (one of the main types of esophageal cancer), larger tumor size, and signs of weakness or difficulty swallowing after initial treatment were associated with a higher risk of recurrence. Patients whose cancer did return could often be treated successfully. These findings may help guide follow-up care, identify high-risk patients early, and reduce unnecessary surgeries, improving quality of life while maintaining good outcomes. They also provide a basis for future research to refine personalized treatment strategies for esophageal cancer.

## 1. Introduction

Esophageal cancer (EC) is a highly lethal malignancy, with over 600,000 newly diagnosed patients and 540,000 deaths annually worldwide. Its incidence continues to rise, with current data projections predicting an even steeper increase in the future [1,2,3,4]. There is an ongoing discussion about the optimal treatment for this cancer entity. The current standard of care for curative treatment of patients with locally advanced but non-metastatic EC consists of a multimodal approach that includes neoadjuvant therapy followed by esophagectomy. According to current guidelines, the treatment for EC consists of either combined radiochemotherapy (CROSS protocol: weekly chemotherapy with carboplatin and paclitaxel with concurrent radiotherapy up to 41.4 Gy over 5 weeks) or chemotherapy alone (FLOT protocol: four preoperative and four postoperative 2-week cycles of 5-fluorouracil, leucovorin, oxaliplatin, and docetaxel) [5,6,7,8,9,10]. However, esophagectomy is associated with high morbidity including pulmonary complications (e.g., pneumonia, pleural effusion), anastomotic leakage or stricture, cardiovascular events (e.g., atrial fibrillation, myocardial infarction), wound infection, and chylothorax, with overall complication rates of 40–60% and severe complications (Clavien–Dindo grade ≥ III) in 15–30% of patients [11,12]. Although mortality rates following esophagectomy have decreased from 30% in the 1970s to about 5% in current studies, morbidity remains high, with reported rates up to 50% [11,12,13,14,15]. Furthermore, factors that increase the risk of developing EC, such as smoking, high alcohol intake, and poor nutritional status, are also associated with technical difficulties during esophagectomy and delayed healing thereafter. Median overall survival following esophagectomy ranges between approximately 3.5 and 4.5 years, with substantial variability depending on cancer characteristics and patient-specific factors [16,17]. Conversely, full recovery from esophagectomy and perioperative (radio)chemotherapy often requires 1.5 to 2 years, and in many cases, patients may never fully regain their preoperative quality of life [18,19,20].

An organ-sparing approach with a “watchful waiting” (WW) strategy has gained importance in recent years. In this concept, after neoadjuvant radiochemotherapy and no signs of cancer persistence at restaging—clinical complete response (cCR)—a close follow-up with 3 monthly clinical, endoscopic and radiographic exams is conducted instead of esophagectomy. A WW strategy in EC remains a topic of ongoing debate, with several studies reporting promising results in selected patient groups [21,22,23,24,25].

The advantages of a WW approach lie in avoiding the potential short- and long-term complications of esophagectomy. However, a significant percentage of patients experience cancer recurrence. Recent results from the randomized SANO trial report recurrence rates of over half of these patients, with 48% having local recurrence and about 17% developing distant metastases [25].

In case of cancer recurrence, salvage surgery has proven to be reasonably safe and feasible. Some series have shown that esophagectomy in these instances is associated with increased complication rates, but still promising outcomes [26,27,28]. Due to these increased risks, it is recommended that patients be carefully evaluated before being selected for the WW approach.

The role of restaging is crucial as the final decision on whether patients can undergo standard trimodal therapy with esophagectomy or a watchful waiting (WW) approach is made at this point. It typically involves endoscopic examination with biopsies, imaging, and clinical evaluation to assess the clinical response. However, this evaluation is challenging and can sometimes result in false negative results. The Pre-SANO trial demonstrated that standard restaging using endoscopy, routine biopsies, and fine-needle aspiration resulted in a false-negative rate of approximately 31%. However, the sensitivity of restaging could be improved by combining multiple diagnostic modalities, including bite-on-bite biopsies, endoscopic ultrasound (EUS), and PET-CT imaging [29]. Ongoing research aims to improve the detection of cancer response through advancements in imaging and biochemical testing [29,30,31]. Some studies have indicated better outcomes for patients with squamous cell carcinoma compared to those with adenocarcinoma undergoing WW [32]. In a secondary analysis of the SANO trial, higher nodal stage at baseline was identified as a risk factor for recurrence; however, the authors emphasized the need for additional data to better define reliable predictive factors [33]. Overall, data on additional clinical risk factors predicting cancer recurrence are limited. This study aims to identify independent clinical factors associated with the recurrence of EC based on data from patients undergoing WW over the past decade

## 2. Materials and Methods

### 2.1. Patient Selection and Data Collection

Prospectively recorded data (according to our hospital standard for the national high-specialized medicine (HSM) surgery database) of patients diagnosed with EC and neoadjuvant radiochemotherapy from January 2014 to December 2022 were extracted from hospital medical records and evaluated retrospectively until December 2024, to ensure a follow-up period of at least 2 years. All patients who underwent WW were included for further analysis. Exclusion criteria were patients without signed general consent, patients not undergoing WW, EC other than adeno- or squamous cell carcinoma, (oligo-)metastatic disease, and synchronous other malignant cancer.

### 2.2. Treatment

Diagnosis of EC was confirmed with upper endoscopy and biopsies. Staging involved endoscopic ultrasound (EUS), thoracoabdominal computed tomography (CT) and FGD18-positron emission tomography (PET). All cases were discussed at an interdisciplinary case conference, after which the neoadjuvant RCT was initiated. Neoadjuvant RCT consisted of radiation with 50.4 Gy and a combination of carboplatin and paclitaxel, following an adapted CROSS protocol [5,34]. Radiation fields included the primary tumor and regional lymphatic drainage areas and were adapted to tumor location, while the total dose and treatment principles remained consistent across all patients. Six weeks after completion of RCT, a restaging, which included upper endoscopy and biopsies, EUS, CT and clinical evaluation, was performed. In case of cCR, defined as no evidence of vital tumor cells in restaging biopsies and no aspects of local or distant progression in CT scan, both surgical resection and a watchful waiting strategy were considered. The final treatment decision was made after interdisciplinary discussion and shared decision-making with the patient, taking into account the overall clinical assessment and patient preference.

### 2.3. Follow-Up

WW consisted of a standardized follow-up protocol for all patients, including upper endoscopies with biopsies and EUS every three months, and CT every six months. In case of suspected EC recurrence, defined as vital tumor cells in esophageal biopsy or signs of local or distant tumor growth in CT or EUS, a complete staging was performed, and further management was discussed at the interdisciplinary case conference and with the patient.

Recurrence was classified into local, regional, and distant categories. Local recurrence was confined to the esophagus without lymph node involvement, whereas regional recurrence involved both the esophagus and the regional lymph nodes. Distant recurrence referred to metastatic disease.

If recurrence was local or regional, patients were addressed with curative intent. Treatment consisted of esophagectomy, or, in selected cases of local recurrence without nodal involvement, endoscopic submucosal dissection (ESD). Esophagectomy was performed as an open extended transhiatal esophagectomy with en-bloc lymphadenectomy in early study years or as minimally invasive (laparo- and thoracoscopic) McKeown or Ivor-Lewis esophagectomy with en-bloc lymphadenectomy in later years. Systemic therapy was initiated in case of distant recurrence, when a patient declined further procedures or had a clinical condition that precluded interventional or surgical measures.

### 2.4. Statistical Analysis

Data were analyzed using statistical software (IBM SPSS Statistics 28). Descriptive statistics were applied to summarize demographic characteristics, clinical variables, and outcomes. Continuous variables are presented as means ± standard deviations (SD) or medians with interquartile ranges (IQR), depending on their distribution. Normality of distribution was assessed using histograms, skewness and the Shapiro–Wilk test. Categorical variables are expressed as frequencies and percentages.

Differences between groups were assessed using appropriate statistical tests, such as the Mann-Whitney U test for continuous variables and Fisher’s exact test for categorical variables. A *p*-value < 0.05 was considered statistically significant. Significant variables were further tested in uni- and multivariable logistic regression to minimize confounding effects and prove independence.

## 3. Results

### 3.1. Baseline Data and Patterns

Out of 458 patients with EC and neoadjuvant radiochemotherapy, 50 patients met the inclusion criteria and were selected for further analysis.

Among these, 8 (16%) had local tumor growth (no positive lymph nodes detected in EUS and/or (PET-)CT scan) and 39 (78%) had positive lymph nodes. In a small number (*n* = 3, 6%), the lymph node state could not be assessed completely due to a stenosis that could not be passed with the endosonographic device.

**Figure 1:**
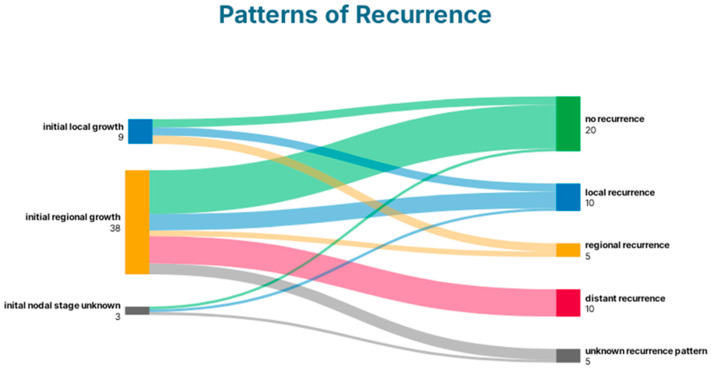
Patterns of recurrence following definitive radiochemotherapy.

30 patients (60%) experienced cancer recurrence during the surveillance period. Most recurrences (*n* = 15, 50%) were local or regional and therefore potentially addressable in curative intention; 10 (33%) had metastatic disease. 5 patients (17%) denied further analysis of their recurrence and chose best supportive care. Patients with initially local growth developed no distant recurrence, while regional recurrence was infrequent in general (Figure 1).

Recurrence occurred after a median of 202 days (IQR 161) after restaging. However, there was no statistically significant difference in time to recurrence if patients had initial local or regional growth (146 days, IQR 116.5 vs. 261.5 days, IQR 245.5, *p* = 0.117).

### 3.2. Baseline Patient and Tumor Characteristics at Staging and Restaging as Risk Factors for Recurrence

Of the 50 patients undergoing a WW-approach, 80% were male, and the median age was 69.0 years (IQR 13.3). They presented with dysphagia in 80% and weight loss in 42%. Patients developing recurrence presented in a slightly worse clinical condition at staging, represented by a median Karnofsky Score of 80% vs. 90%. Otherwise, there were no statistically significant differences between the groups Table 1.

Tumor characteristics are listed in Table 2. In univariate analysis, EC type (*p* = 0.042), tumor circumference at diagnosis, or rather, luminal circumference greater than 50% (*p* = 0.013) were significant risk factors for cancer recurrence. Neither location, preoperative stage, nor nodal state had a significant impact on recurrence.

At restaging, only subjective factors were associated with a significantly elevated risk for recurrence: reported fatigue (*p* = 0.03), dysphagia (*p* = 0.05) and deterioration of general condition (*p* < 0.01) (Table 3).

### 3.3. Uni- and Multivariate Logistic Regression

All variables that showed as significant in univariate analysis were tested by logistic regression to assess their amount of influence as negative predictive factors (Table 4). Tumor type (adenocarcinoma), circumferential extent > 50%, as well as dysphagia and fatigue at restaging, showed significant and realistic odds ratios and were further tested for multivariate analysis.

Given the limited sample size and number of events, the number of covariates included in the multivariable model was deliberately restricted to reduce the risk of overfitting. The selected factors were adjusted for common confounders, including age, sex, BMI, comorbidities, and tumor stage. All predictors remained statistically significant after adjustment. 

Furthermore, they were tested against each other to predict independence. Only adenocarcinoma as a tumor type showed an independent significant influence (*p* = 0.022), while the others missed the 0.05 benchmark shortly. However, partially wide confidence intervals were noted, probably due to the relatively small sample size.

## 4. Discussion

This study analyzes predictive factors and patterns of recurrence in patients with esophageal cancer who underwent a watchful waiting strategy following neoadjuvant radiochemotherapy. Tumor involvement of more than 50% of the esophageal circumference on pre-treatment endoscopy, adenocarcinoma, and the presence of dysphagia, fatigue, or deterioration of general condition at restaging were identified as independent risk factors for cancer recurrence. There were no distant recurrences in patients with initial local growth. In patients with initial regional growth (adjacent positive lymph nodes), 16% had local and 26% distant recurrence.

Although a watchful waiting concept is not yet considered standard of care in EC, it is gaining increasing importance in clinical practice as such a strategy has been proven to be safe and feasible across several studies [21,22,23,24,25]. Most notably, recent results from the prospective, multicenter SANO trial support the clinical applicability of this approach, suggesting that more centers will adopt WW in the future [25]. However, rates of cancer recurrence remain high. In our cohort, a recurrence rate of 60% was observed, in line with previously published data [22,25]. While this is higher than the recurrence risk after standard surgical resection, a direct comparison is limited because surgery removes the esophagus and potentially minimal tumor residual entirely, but induces substantial operative morbidity. WW allows almost 50% of patients to avoid surgery altogether, highlighting the trade-off between a higher recurrence rate and the benefits of organ preservation. Evidence from the literature indicates that health-related quality of life is significantly improved with an organ-preserving WW strategy compared with upfront surgery, without compromising long-term survival [35,36]. Most recurrences in our cohort were local and potentially amenable to curative salvage esophagectomy, though such procedures carry substantial risks [26,27,28]. These findings emphasize that careful patient selection, comprehensive patient education, and close surveillance are essential, and that the acceptability of WW depends on balancing recurrence risk against the morbidity of surgery [37]. Early detection of recurrence is crucial to prevent progression to systemic disease and the subsequent need for systemic, palliative treatment.

Currently, there is no validated or standardized follow-up protocol for patients undergoing WW, and monitoring strategies vary significantly between institutions. Identifying predictors of recurrence can contribute to the development of consensus-based surveillance protocols, enabling the stratification of patients according to risk and facilitating more individualized follow-up plans. For example, a secondary analysis of the SANO trial evaluated parameters predicting recurrence and identified a higher nodal stage at baseline as a risk factor, highlighting the potential value of incorporating both clinical and pathological factors into surveillance strategies [33]. Similarly, our previous work demonstrated that early detection of recurrence enables curative-intent treatment and favorable outcomes, emphasizing the clinical relevance of identifying patients at increased risk [38]. Such efforts could improve clinical outcomes and promote patient-centered care.

It is well established that squamous cell carcinoma tends to respond more favorably to neoadjuvant radiochemotherapy, whilst adenocarcinoma is more prone to recurrence. Our findings are consistent with this, identifying adenocarcinoma as a significant independent risk factor for recurrence. Nevertheless, we do not suggest that WW should be categorically avoided in these patients. Although evidence remains limited, the SANO trial demonstrated favorable outcomes for selected patients with adenocarcinoma undergoing WW [25]. Such cases require careful patient selection, close monitoring, and comprehensive patient education. In our patients, the time to recurrence in this subpopulation was 16.8 months. These patients should be monitored with increased vigilance, and both clinicians and patients must be informed of the elevated risk.

Another negative prognostic factor identified was tumor involvement of more than 50% of the esophageal circumference at staging endoscopy. This might be a surrogate for the progression of the disease. Further, lymphadenopathy at the time of staging did not play a role, nor did longitudinal tumor spread.

Interestingly, patient-reported symptoms and general clinical condition at restaging emerged as strong predictors of recurrence. Specifically, dysphagia, fatigue, and reduced general condition were all associated with a significantly higher likelihood of recurrence. This might be caused by minimal residual or systemic disease that is undetectable by endoscopic, histological, or radiological means. Importantly, at the time of assessment, all patients had no radiologic or histologic evidence of recurrence, making these clinical factors the only detectable indicators of increased risk. This observation is clinically valuable, as it relies on routine history-taking and physical examination rather than expensive or invasive diagnostic tools, making it an easily accessible method for risk stratification.

Furthermore, this highlights the limitations of currently available methods for assessing tumor response to radiochemotherapy, as well as the absence of robust, clinically meaningful biomarkers. Noordman et al., in the preSANO trial, emphasized the necessity of integrating multiple diagnostic modalities to improve the accuracy of staging and restaging. They reported relatively low sensitivities for individual techniques such as endoscopy with (bite-on-bite-) biopsy, endoscopic ultrasound, and (PET-) CT imaging [29]. Also, unlike other gastrointestinal malignancies, reliable tumor markers for EC are currently lacking. Several studies have investigated emerging approaches, including liquid biopsy, for their potential prognostic and diagnostic utility, yielding promising preliminary results. Specifically, several studies have investigated the utility of circulating tumor DNA (ctDNA) in the context of restaging and the prediction of pathological complete response. Although these preliminary data appear promising, the current sensitivity and specificity of ctDNA-based assays remain inadequate for their routine implementation in standard restaging procedures [39,40,41]. In the future, multimodal predictive models that integrate clinical, radiological, endoscopic, and laboratory parameters hold promise for enhancing personalized surveillance strategies in esophageal cancer management.

In this series, we observed that one-third developed distant recurrence, while another 33% experienced limited local recurrence, and 17% presented with regional lymph node involvement. In 17% of cases, the recurrence pattern could not be determined due to the patients’ decision to forego further diagnostic evaluation following the detection of recurrence. Due to the relatively small sample size, we were unable to identify specific risk factors associated with distant versus locoregional recurrence. We strongly advocate for future studies addressing this question, as they may facilitate more individualized treatment regimes, including tailored neoadjuvant regimens (e.g., radiochemotherapy, chemotherapy or even in combination with immunotherapy), individualized and improved restaging protocols, and follow-up strategies guided by probable recurrence patterns.

This study has several limitations. The retrospective and non-randomized design introduces potential biases, despite using multivariate analysis to mitigate them. Due to the retrospective design, detailed information on the decision-making process for individual patients is limited. Selection for the WW strategy in patients with cCR likely involved a combination of clinical assessment, physician judgment, and patient preference. Similarly, for patients judged to have achieved a cCR but who underwent surgery, the specific factors influencing the decision cannot be fully determined retrospectively. Consequently, selection bias cannot be excluded and should be considered when interpreting the findings. Treatment-related adverse events associated with chemoradiotherapy were not systematically recorded in our retrospective dataset and therefore could not be included in the morbidity assessment, which in this study primarily reflects postoperative complications.

The relatively small sample size also limited further subgroup analysis and methods like propensity score matching. In addition, the limited number of events and cohort size restricted statistical power, particularly for the identification of independent predictive factors. Although the multivariable models were carefully specified, the potential risk of overfitting cannot be fully excluded. The broad confidence intervals observed in the multivariate analysis highlight these limitations, and although the results were statistically significant, they should be interpreted with caution and considered hypothesis-generating, requiring validation in larger cohorts before implementation in clinical practice. Furthermore, follow-up in our study was limited to a two-year period. This decision was made to include a sufficiently large and representative sample. In addition, both our data and the existing literature indicate that most cancer recurrences in a WW strategy to occur within the first two years after restaging. Therefore, we consider this follow-up period to be clinically appropriate and unlikely to have missed a substantial number of late recurrences.

Additionally, given the subjective nature of some identified predictive factors, such as patient-reported symptoms, there is an inherent risk of observer bias and unmeasured confounding. Although data were collected systematically during routine clinical assessments, inter-observer variability cannot be ruled out. Thus, prospective multicenter studies with larger patient populations are warranted to confirm our findings.

Lastly, we included only patients who received preoperative RCT, as the WW strategy is primarily applied in this subgroup, and current evidence on WW following chemotherapy alone remains insufficient. Recent data indicate a shift in perioperative treatment of EC toward chemotherapy based on the FLOT protocol, rather than the CROSS regimen [42,43]. The potential role of an organ-preserving approach within this evolving treatment paradigm warrants further investigation.

## 5. Conclusions

In this study of fifty patients in a WW approach to EC, 60% developed recurrence after a median of 202 days. Recurrence was local or regional in 50% and distant in 33%. Predictive factors for recurrence were adenocarcinoma histology, tumor involvement of more than 50% of the esophageal circumference at staging, and the presence of dysphagia, fatigue, or deteriorated general condition at restaging.

## Figures and Tables

**Table 1:** Baseline characteristics of patients at diagnosis.

	Total	Tumor Recurrence	No Recurrence	*p*-Value
Number	*n* = 50	*n* = 30 (60.0)	*n* = 20 (40.0)	
Age	69.0 (13.3)	70.0 (13.0)	66.0 (14.5)	0.204
Gender, male	40 (80.0)	26 (86.7)	14 (70.0)	0.171
Body Mass Index ^1^	24.7 (6.8)	25.0 (7.7)	23.6 (6.1)	0.801
Weight loss in kg ^1^	4.0 (8.0)	4.0 (8.0)	3.0 (7.5)	0.681
Charlson Comorbidity Score ^1^	5.5 (2.3)	6.0 (3.0)	5.0 (3.0)	0.056
Initial Karnofsky Score ^1^	80.0 (10.0)	80.0 (15.0)	90.0 (10.0)	0.048
Initial NRS ^1^*	3.0 (2.0)	3.0 (2.0)	3.0 (1.5)	0.193
*Risk factors*				
Nicotine	31/48 (64.6)	19/29 (65.5)	12/19 (63.2)	1.000
Alcohol	27/48 (56.3)	15/29 (51.7)	12/19 (63.2)	0.555
*Symptoms*				
Dysphagia	40 (80.0)	24 (80.0)	16 (80.0)	1.000
Odynophagia	10 (20.0)	6 (20.0)	4 (20.0)	1.000
Reflux	13 (26.0)	9 (30.0)	4 (20.0)	0.522
Weight loss in kg	21 (42.0)	16 (53.3)	5 (25.0)	0.079
Deterioration of GC *	6 (12.0)	5 (16.7)	1 (5.0)	0.381
Regurgitation	6 (12.0)	5 (16.7)	1 (5.0)	0.381
Retrosternal pain	6 (12.0)	3 (10.0)	3 (15.0)	0.672

Values are numbers (percentages) unless indicated. ^1^ values are medians (interquartile ranges) * NRS: nutritional risk score. GC: general condition.

**Table 2:** Tumor characteristics.

	Total	Tumor Recurrence	No Recurrence	*p*-Value
Number	*n* = 50	*n* = 30 (60.0)	*n* = 20 (40.0)	
Type				
- Adeno	22 (44.0)	17 (56.7)	5 (25.0)	0.042
- Squamous	28 (56.0)	13 (43.3)	15 (75.0)	0.042
Margin proximal ^1^	29.5 (8.3)	30.0 (8.3)	28.0 (9.5)	0.558
Margin distal ^1^	35 (11.5)	36.0 (10.3)	30.0 (9.5)	0.444
Lengtht ^1^	4.5 (4.3)	5.5 (5.3)	3.5 (2.8)	0.162
Circumference in % ^1^	60.0 (70.0)	82.5 (50.0)	40.0 (25.0)	0.010
Circumference > 50%	26/45 (57.8)	20/27 (74.1)	6/18 (33.3)	0.013
Stenosis at diagnosis	23 (46.0)	16 (53.3)	7 (35.0)	0.254
*Extent of cancer*				0.876
Initial local growth	9 (18.0)	6 (20.0)	3 (15.0)	0.724
Initial regional growth	38 (76.0)	22 (73.3)	16 (80.0)	0.740
Initial nodal state unknown	3 (6.0)	2 (6.7)	1 (5.0)	1.000
*Pretherapeutic Stage*				
T1	3/48 (6.3)	2/29 (6.9)	1/19 (5.3)	1.000
T2	9/48 (18.8)	4/29 (13.8)	5/19 (26.3)	0.451
T3	32/48 (66.7)	21/29 (72.4)	11/19 (57.9)	0.357
T4a	3/48 (6.3)	2/29 (6.9)	1/19 (5.3)	1.000
T4b	1/48 (2.1)	-	1/19 (5.3)	0.396
N0	8/47 (17.0)	5/28 (17.9)	3/19 (15.8)	1.000
N1	22/47 (46.8)	11/28 (39.3)	11/19 (57.9)	0.246
N2	13/47 (27.7)	10/28 (35.7)	3/19 (15.8)	0.189
Pretherapeutic UICC * Stadium				
Stadium I	1/48 (2.1)	1/29 (3.4)	0 (0)	1.000
Stadium II (a + b)	10/48 (20.8)	4/29 (13.8)	6/19 (31.6)	0.164
Stadium III	32/48 (66.7)	23/29 (79.3)	9/19 (47.4)	0.031
Stadium Iva	5/48 (10.4)	1/29 (3.4)	4/19 (21.1)	0.072

Values are numbers (percentages) unless indicated. ^1^ values are medians (interquartile ranges) * UICC: Union Internationale Contre le Cancer.

**Table 3:** Findings at restaging.

	Total	Tumor Recurrence	No Recurrence	*p*-Value
Number	*n* = 50	*n* = 30 (60.0)	*n* = 20 (40.0)	
*Symptoms*				
Dysphagia	29 (58.0)	21 (70.0)	8 (40.0)	0.045
Odynophagia	6 (12.0)	3 (10.0)	3 (15.0)	0.672
Weight Loss	12 (24.0)	10 (33.3)	2 (10.0)	0.091
Deterioration of GC *	11 (22.0)	11 (36.7)	0 (0)	0.002
Fatigue	14 (28.0)	12 (40.0)	2 (10.0)	0.026
EGD * findings				
Endoscopically non-passable stenosis	10 (20.0)	8 (26.7)	2 (10.0)	0.279
Ulcus	24 (48.0)	15 (50.0)	9 (45.0)	0.779
Scar	10 (20.0)	6 (20.0)	4 (20.0)	1.000
*Histopathology*				
Inflammation	10 (20.0)	8 (26.7)	2 (10.0)	0.279
Reactive changes	24 (48.0)	15 (50.0)	9 (45.0)	0.779
*Intestinal metaplasia*	10 (20.0)	6 (20.0)	4 (20.0)	1.000
Fibrosis	10 (20.0)	8 (26.7)	2 (10.0)	0.279
Atypical cells	24 (48.0)	15 (50.0)	9 (45.0)	0.779
Ulcerations	10 (20.0)	6 (20.0)	4 (20.0)	1.000
Regression of tumor in (PET-)CT	32/46 (69.6)	21/28 (75.0)	11/18 (61.1)	0.345
Regression of lymph nodes in (PET-)CT	22/37 (59.5)	13/22 (59.1)	9/15 (60.0)	1.000

Values are numbers (percentages) unless indicated. * GC: general condition, EGD: esophagogastroduodenoscopy.

**Table 4:** Uni- and multivariate regression.

	HR Univ.	95% CI Univ.	*p*-Value Univ.	HR Multiv. ^1^	95% CI Multiv. ^1^	*p*-Value Multiv. ^1^	HR Multiv. ^2^	95% CI Multiv. ^2^	*p*-Value Multiv. ^2^
Type adenocarcinoma	3.92	1.13–13.60	0.031	5.49	1.10–27.29	0.038	25.14	1.60–393.72	0.022
Circumference > 50%	5.71	1.55–21.06	0.009	25.19	2.69–236.17	0.005	4.69	0.75–29.48	0.099
Dysphagia at restaging	3.50	1.07–11.8	0.039	4.36	1.04–18.27	0.044	12.92	0.99–169.38	0.051
Fatigue at restaging	6.00	1.17–30.73	0.032	9.47	1.39–64.62	0.022	12.19	0.92–161.39	0.058

^1^ multivariate adjusted for co-founders’ age, gender, BMI, Charlson comorbidity index and tumor stage. ^2^ multivariate adjusted for other significant factors.

## Data Availability

The data supporting the findings of this study are available from the corresponding author upon reasonable request. Due to patient privacy and institutional regulations, the data are not publicly available.

## Abbreviations

The following abbreviations are used in this manuscript:

cCR	Clinical complete response
CT	Computed tomography
ctDNA	Circulating tumor DNA
EC	Esophageal cancer
EGD	Esophagogastroduodenoscopy
EUS	Endoscopic ultrasound
GC	General condition
HSM	High-specialized medicine
IQR	Interquartile ranges
NRS	Nutritional risk score
PET	PDG18-positron emission tomography
RCT	Radiochemotherapy
SD	Standard deviation
UICC	Union Internationale Contre le Cancer
WW	Watchful Waiting
